# Directional Theta Coherence in Prefrontal Cortical to Amygdalo-Hippocampal Pathways Signals Fear Extinction

**DOI:** 10.1371/journal.pone.0077707

**Published:** 2013-10-24

**Authors:** Jörg Lesting, Thiemo Daldrup, Venu Narayanan, Christian Himpe, Thomas Seidenbecher, Hans-Christian Pape

**Affiliations:** 1 Institute of Physiology 1, Westfälische Wilhelms-University, Münster, Germany; 2 Otto Creutzfeldt Centre for Cognitive and Behavioral Neuroscience, Westfälische Wilhelms-University, Münster, Germany; 3 Institute for Computational and Applied Mathematics, Westfälische Wilhelms-University, Münster, Germany; University of Alberta, Canada

## Abstract

Theta oscillations are considered crucial mechanisms in neuronal communication across brain areas, required for consolidation and retrieval of fear memories. One form of inhibitory learning allowing adaptive control of fear memory is extinction, a deficit of which leads to maladaptive fear expression potentially leading to anxiety disorders. Behavioral responses after extinction training are thought to reflect a balance of recall from extinction memory and initial fear memory traces. Therefore, we hypothesized that the initial fear memory circuits impact behavioral fear after extinction, and more specifically, that the dynamics of theta synchrony in these pathways signal the individual fear response. Simultaneous multi-channel local field and unit recordings were obtained from the infralimbic prefrontal cortex, the hippocampal CA1 and the lateral amygdala in mice. Data revealed that the pattern of theta coherence and directionality within and across regions correlated with individual behavioral responses. Upon conditioned freezing, units were phase-locked to synchronized theta oscillations in these pathways, characterizing states of fear memory retrieval. When the conditioned stimulus evoked no fear during extinction recall, theta interactions were directional with prefrontal cortical spike firing leading hippocampal and amygdalar theta oscillations. These results indicate that the directional dynamics of theta-entrained activity across these areas guide changes in appraisal of threatening stimuli during fear memory and extinction retrieval. Given that exposure therapy involves procedures and pathways similar to those during extinction of conditioned fear, one therapeutical extension might be useful that imposes artificial theta activity to prefrontal cortical-amygdalo-hippocampal pathways that mimics the directionality signaling successful extinction recall.

## Introduction

Precisely timed neuronal activity is crucial for communication within synaptic networks across brain areas, as required for instance for cognitive tasks and integrative behavioral responses [1]–[3]. One example is the synchronization at theta frequencies between amygdala and hippocampus during consolidation of fear memories [4], [5]. Theta synchrony is high between the hippocampal subfield CA1 and the lateral amygdala (LA) during consolidation [5] and reconsolidation [6] of fear memory, and theta synchrony decreases at remote memory stages [7]. In keeping with this, the degree of theta coupling occurring during paradoxical sleep in the basolateral complex of the amygdala (BLA), hippocampus and medial prefrontal cortex (mPFC) predicts the consolidation success of the fear memory [8]. Ventral hippocampal inputs also influence anxiety-related activity in mPFC neurons, and synchronization at theta frequencies is involved in this interaction [9], [10]. From this data it has been inferred that synchronized theta oscillations selectively recruit neurons to form a fear memory trace, by providing spatiotemporal codes for temporal compression from the rather long time scale of learned behavior down to the milliseconds timescale required for synaptic plasticity and orchestra molecular cascades of memory stabilization [4], [11]–[13].

Adaptive mechanisms are of critical importance for the regulation of emotions, in that they allow the individual to control otherwise stereotyped reactions to emotionally salient stimuli. One prominent example is extinction, a form of inhibitory learning that allows adaptive control of emotional responses like conditioned fear [14]–[16]. A deficit in fear extinction leads to maladaptive expression of fear and has been proposed to underlie mood and anxiety disorders [15], [17]–[19]. Furthermore, exposure therapy that is clinically used to treat anxiety disorders is based on an approach used to extinguish conditioned fear responses in the laboratory. In both cases, the subject is repeatedly presented with the fear conditioned stimulus (CS) in the absence of the threat or unconditioned stimulus (US), resulting in a decline of CS-evoked fear responses. Much evidence indicates that this form of learning depends on the development of a new associative memory that competes with the initial fear memory for control of behavior, although there is also evidence that weakening of the initial CS-US association is involved under certain circumstances [14], [20]. Synchronized neuronal activity at theta frequencies seems to be a crucial element to spatiotemporally coordinate brain areas also during fear extinction, as is indicated by the rebound of theta synchrony in CA1/LA/mPFC circuits during extinction recall [21]. However, correlated neuronal activity in the amygdala and the dorsal anterior cingulate cortex (dACC), a brain area thought to regulate expression of learned fear responses, has been found to predict the persistence of aversive memories and thereby their resistance to extinction [22].

While theta synchrony thus seems to represent a principle mechanism to recruit neuronal subpopulations across brain areas in a fear memory trace during consolidation and recall, its impact on fear extinction is not well understood. Specifically, conditioned fear can be either successfully extinguished or it may persist, and behavioral responses after extinction training are thought to reflect a balance of recall from extinction memory and initial fear memory traces. Therefore, we hypothesized that the initial fear memory circuits involving CA1/LA/mPFC impact behavioral fear after extinction, and that the dynamics of theta synchrony in these pathways predict the individual fear response. We addressed this by combining simultaneous multi-channel local field and unit recordings in CA1, LA and infralimbic region of the mPFC (IL-PFC) in behaving mice subjected to Pavlovian auditory fear conditioning and extinction training.

## Materials and Methods

### Animals

A total of 13 male C57Bl/6J mice (8–12 weeks of age) were used in accordance with the regulations of the German law and as approved by the local authorities (Bezirksregierung Münster, AZ 50.0835.1.0, G 53/2005). Animals were kept in a 12 hour light-dark cycle, provided with food and water ad libitum and included in the experiments at 8 to 12 weeks of age.

### Electrode Implantation

**Figure 1:**
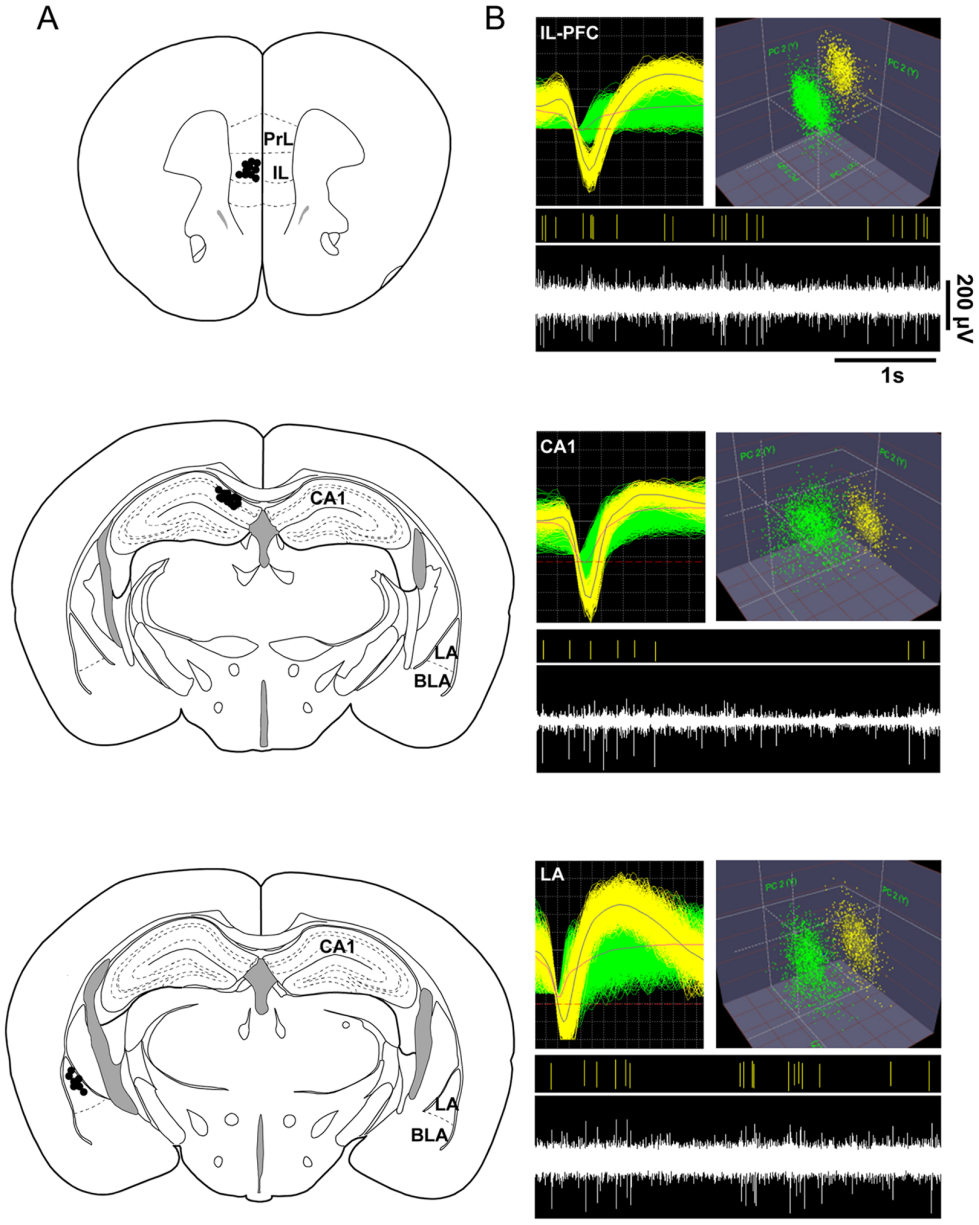
Verification of recording sites and isolation of single units. (A) Schematic representation of electrode locations in the infralimbic prefrontal cortex (IL-PFC), hippocampal CA1, and lateral amygdala (LA). Black dots mark recordings sites. (B) Representative examples of extracellular waveform sorting in IL-PFC, CA1 and LA. The top images show superimposed waveforms of two simultaneously recorded neurons and cluster analysis, formed in three-dimensional space after applying principal component analysis. The bottom images display the sorted unit (yellow) of the continuous signal below. PrL, prelimbic region of mPFC; IL, infralimbic region of mPFC; BLA, basolateral amygdala; LA lateral amygdala.

For recording of neuronal and network activities (spontaneous extracellular local field potential (LFP) and unit activities) in freely behaving mice, micro-wire arrays (MWA, 1 array, 8 electrodes and one reference/array per brain region; Stablohm 650; California Fine Wire) were implanted under stereotaxic control (David Kopf Instruments). The tip of each wire was gold-plated by passing a cathodal current of 1 µA while wires were submerged in a gold solution to reduce the impedance to a range of 150–300 kΩ. Under deep pentobarbital anesthesia (50 mg/kg i.p.), supplemented by subcutaneous injection of Carprofen (Rimadyl; 5 mg/kg), electrodes were implanted in the left hemisphere at the following stereotactical coordinates [23]: CA1: −1.94/1/1.25 mm, LA: −2.06/3.25/3.2 mm, and IL-PFC: 1.75/0.3/2.0 mm from bregma. Electrodes were fixated with dental cement. Experiments involved a ground electrode, positioned close to the midline over the cerebellar region (5.8/0.5 mm from bregma) of the right hemisphere. At the end of the experiments animals were killed by an overdose of pentobarbital (100 mg/kg, i.p.), location of recording sites were marked by small electrolytic lesions (1 mA anodal current for 10 s), and brains were rapidly removed and fixed in 4% phosphate-buffered formaldehyde, pH 7.4. Electrode positions were identified in 50 µM cresyl violet counterstained frontal brain sections (Figure 1A).

### Behavioral Paradigm

After 7–10 days of surgical recovery, animals underwent fear conditioning as previously described [21], [24]–[27]. In brief, mice were adapted to the fear conditioning apparatus (TSE, Bad Homburg, Germany) and exposed to a neutral tone presented six times (CS−, 2.5 kHz, 85 dB, 10 s, 20 s inter-stimulus interval (ISI)). This adaptation session was repeated once. Fear conditioning took place the following day and consisted of a tone presented three times [conditioned stimulus (CS+), 10 kHz, 85 dB, 10 s, ISI randomized 10–30 s], each coterminated with a 1 s footshock (scrambled, 0.4 mA). This conditioning session was repeated once 6 h later. The next day, retrieval of fear memory and extinction training was started. Under slight Forene anesthesia (isofluran, 1-chloro-2,2,2-trifluoroethyldifluoromethylether) animals were connected to a swivel commutator of the recording device and after a recovery period of 30 min the experiment started. The protocol involved 6 retrieval sessions (R1–R6, 6 min each, separated by 30 min), in which both the CS− and CS+ were presented (4 times each) in a neutral context. Recall of fear extinction was evaluated on the following day through one session in the extinction context (E; identical to the retrieval sessions; Figure S1). Behavioral expressions (e.g. freezing, risk-assessment, rearing, exploration), were evaluated online by an experienced experimenter and recorded as video file for offline analysis, as necessary. Freezing, an innate defensive behavior defined as complete immobility with the exception of respiratory movements, was taken as a behavioral measurement of fear [28]. In the sessions (R1, R6 and E) freezing time was calculated as percentage during the first CS+ presentation (10 s).

### Electrophysiology

Neuronal activities (LFPs and unit activities) were recorded throughout the experimental procedure. Recorded electrical activities were fed through a Multichannel Amplifier System MAP16 (Plexon Incorp., Dallas, Texas USA), and stored on-line on a personal computer. LFP and unit activity were bandpass filtered at 0.7–154 Hz for LFP, and 100 Hz-13 kHz for unit activities, and sampled at rates of 1 kHz and 40 kHz, respectively. Spikes of individual neurons were sorted by time-amplitude window discrimination and principal component analysis (OfflineSorter, Plexon Inc., Dallas, Texas USA), and verified through quantification of cluster separation (Figure 1B). Electrophysiological analyses (see below) were confined to the entire length of the first CS+ presentation (10 s) in R1, R6 and E. Additionally, as increases in theta coupling have previously been related to conditioned fear behavior [5], [6], [8], [21], electrophysiological analyses were further differentiated to data segments, during which the animals displayed freezing or no freezing while exposed the first presented conditioned stimulus (CS+) of R1, R6 and E.

### Unit Theta Phase Locking

Intrinsic phase-related unit activity was computed by assigning each spike to the theta phase (derived from a Hilbert transform) of the simultaneously recorded, theta frequency-filtered LFP (4–8 Hz) of the same brain area. The resulting mean resultant length vector (MRL) value was computed as a measure of phase-locking strength and significance was determined by Rayleigh’s test for circular uniformity (for details see [29]).

### LFP Related Directed Theta Synchrony

Data were transferred to Matlab for analysis. As described by Adhikari et al. [9], [30], cross-correlations of instantaneous amplitudes of LFP oscillations between all possible pairs of regions (CA1/LA, CA1/IL-PFC, LA/IL-PFC) were performed to determine the position of the correlation peak as an indicator of the directionality (lagging or leading) between oscillating signals in different brain areas. Briefly, this method comprises of four steps: first, LFPs are band-pass filtered (4–8 Hz); second, the instantaneous amplitudes of the filtered signals were calculated; third, these amplitudes were cross-correlated and the lag at which the cross-correlation peak occurs was determined; fourth, the distribution of lags obtained was tested to determine if it differs from zero; a positive value indicated that interactions are driven by one region and negative value indicates that the interaction is driven by the respective other.

### Directional Unit Phase-locking Analysis

By use of custom written Matlab routines (modified from [29], [31]), directional unit phase-locking analysis was conducted as described by Sigurdsson et al. [29]. Briefly, each spike was assigned a theta phase derived from a Hilbert transformation of the theta-frequency filtered LFP (4–8 Hz) recorded simultaneously in the same (*within* region analysis) or the two other brain areas (*across* region analysis). The mean resultant length vector (MRL) value was computed as a measure of phase-locking strength and significance was determined by Rayleigh’s test for circular uniformity. To determine directionality of the significantly phase-locked units, MRL was calculated for 50 different temporal offsets (4 ms bin, from −100 to +100 ms) for each single unit spike train in relation to the theta filtered LFP; directionality was determined by the location of the peak MRL value for cells. Positive values indicate a lag of the unit related to the respective LFP and negative values indicate a lead.

### Statistics

To determine significance of directionality during R1, R6 and E paired Wilcoxon’s signed rank non-parametric test was used. One-way ANOVA was used to compare freezing in R1, R6 and E and to analyze the number of theta phase locked units in R1, R6 and E, followed by Tukey’s test for multiple comparisons. All values are expressed as mean±SEM.

## Results

### Theta Activity in CA1, LA and IL-PFC during Conditioned Fear and Extinction

**Figure 2:**
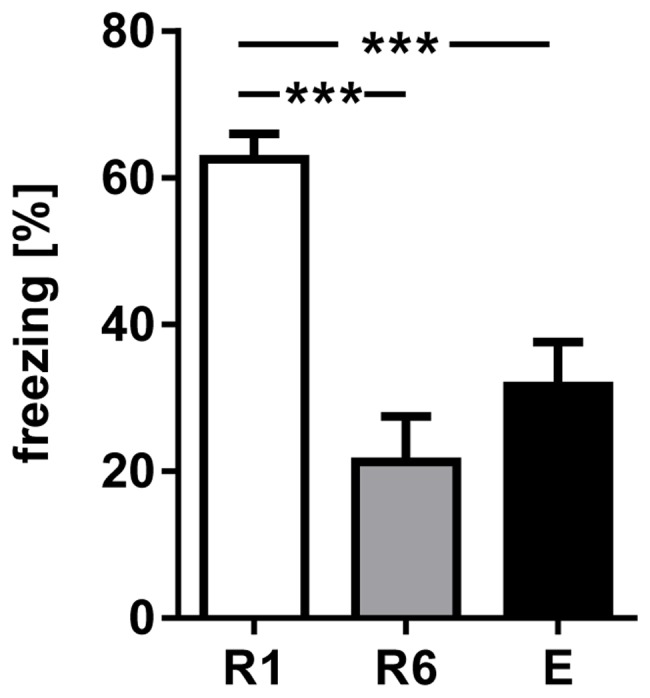
Conditioned freezing in response to presentation of the first CS+ during R1, R6 and E. The fraction of time spent freezing during presentation of the first CS+ significantly declined comparing R1 with R6 and E. Values are mean±SEM; asterisks indicate the significance level: ***,p<0.001 [One-way ANOVA (F_2,36_ = 16.03, p<0.001), followed by Tukey’s post hoc test for multiple comparison], n = 13.

Mice were fear-conditioned using an auditory Pavlovian paradigm (Figure S1) [21]. Conditioned fear (freezing) was analyzed during the first presentations of the conditioned stimulus (CS+) in the first retrieval sessions (R1), at the end of extinction training (R6) and during recall of fear extinction (E) twenty-four hours later. We have focused on the first CS+ presentation, in line with previous studies related to theta oscillations [5]–[7], [21], [25]–[27]. The rational is that freezing behavior has proven to be a good measure of conditioned fear in mice, with the additional consideration that the degree of freezing tends to decline upon successive CS+ retrieval [28]. Animals (n = 13) displayed a high percentage of freezing upon CS+ presentation in R1, which was significantly decreased after extinction training (R6) and during recall of fear extinction (E). One-way ANOVA revealed a significant effect of session (R1, R6 and E) on first CS+ elicited freezing (F_2,36_ = 16.03, p<0.001), and post-hoc multiple comparison (Tukey’s) showed that freezing in R6 and E was significantly decreased compared to R1 (R1, 62.56±3.36%; R6 21.3±6.21%, p<0.001; E, 31.65±5.98%, p<0.001; Figure 2).

**Figure 3:**
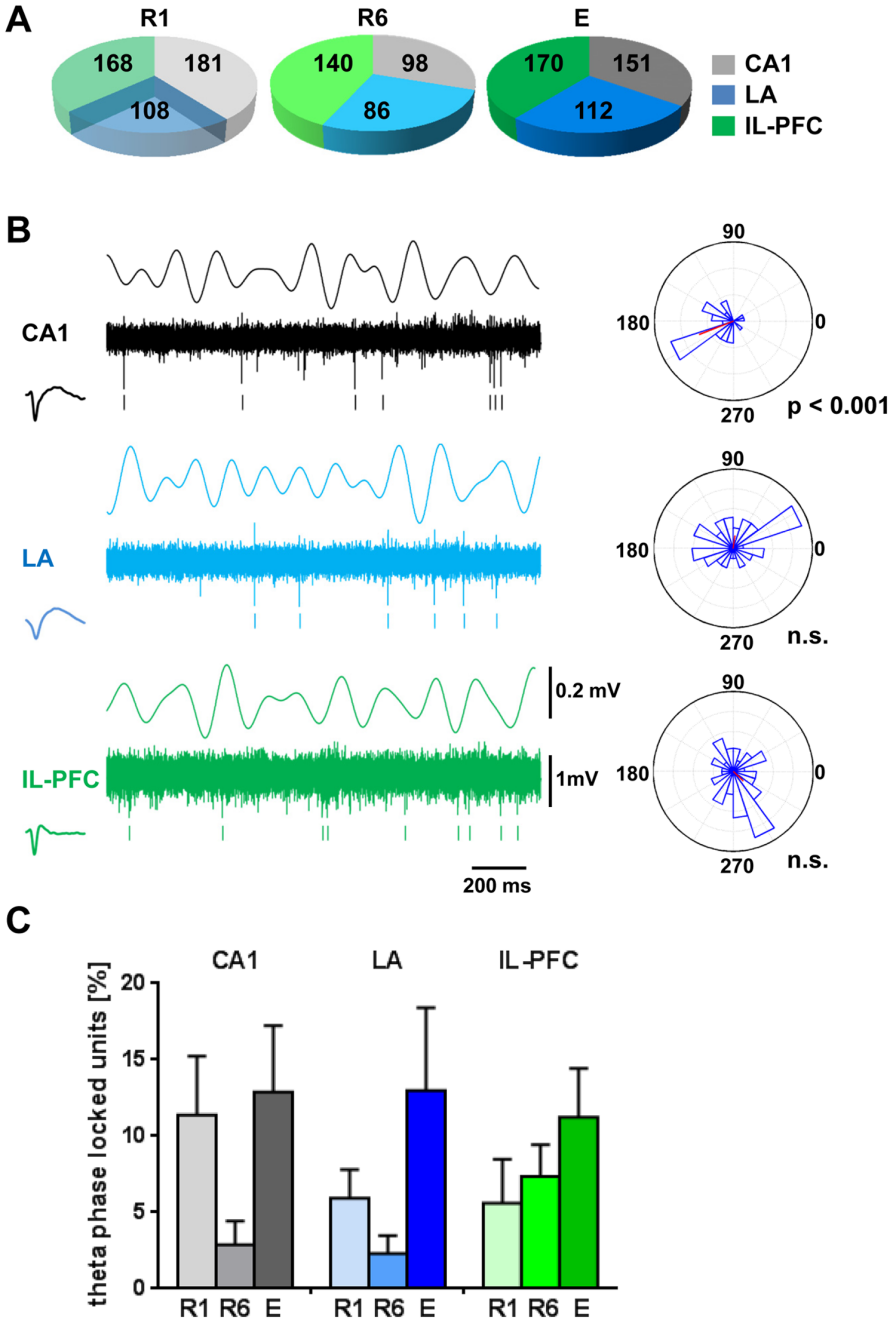
Unit activity in relation to theta LFP during 1CS+ presentation at fear memory retrieval (R1), after extinction training (R6) and at recall of fear extinction (E). Pie chart in (A) summarizes the total number of identified neurons in CA1, LA and IL-PFC. (B) Representative example from one animal during CS+ presentation. The figure shows simultaneously recorded local field potentials (LFP, theta filtered 4–8 Hz), multiple-unit activity (continuous signal), timestamps of identified units in CA1 (black), LA (blue) and IL-PFC (green), and the circular phase distribution of the identified units in relation to the theta filtered LFP (4–8 Hz). Rayleigh’s test for circular uniformity revealed significant phase locking of the CA1 unit to the intrinsic CA1 filtered LFP. (C) Percentage of cells with significant phase-locked unit firing to theta in each structure during R1, R6 and E. One-way ANOVA revealed no significant effect of session (R1, R6 and E) for CA1, LA and IL-PFC phase-locked neurons. n.s., non-significant phase locking (Rayleigh’s test for circular uniformity).

Individual neurons were sorted by time-amplitude window discrimination and principal component analysis (Figure 1B) that had been recorded in CA1, LA and IL-PFC during R1, R6 and E (R1∶181, 108 and 168 identified neurons in CA1, LA and PFC, respectively; R6∶98, 86 and 140; E: 151, 112 and 170; Figure 3A). Next, we performed unit phase locking analysis of each identified neuron in response to the intrinsic theta filtered LFP (4–8 Hz) in the respective brain area during the 1CS+ presentation in R1, R6 and E. An example illustrating unit activity in one individual animal is illustrated in Figure 3B. Rayleigh’s test for circular uniformity revealed that 11.39±3.85% (R1), 2.87±1.55% (R6) and 12.87±4.37% (E) of CA1, 5.94±1.87% (R1), 2.31±1.18% (R6) and 12.97±5.44.% (E) of LA, and 5.61±2.87% (R1), 7.36±2.08% (R6) and 11.25±3.18% (E) of IL-PFC neurons were significantly phase-locked (p<0.05) to the intrinsic LFP (Figure 3C). One-way ANOVA revealed no significant effect of session (R1, R6 and E) for CA1, LA and IL-PFC phase-locked neurons.

### Directional Analysis of Theta Dynamics

To elucidate how fear memory-related coupled theta activities are temporally coordinated in the CA1/LA/IL-PFC network, we used two well established strategies for data analysis: cross-correlation of instantaneous LFP amplitudes, to estimate directionality between brain areas [30], and directional unit phase-locking analysis [29], [32]. As robust theta coherence consisted in CA1/LA/IL-PFC throughout R1, R6 and E (Figure S2), and increases in LFP theta synchrony in the CA1/LA/IL-PFC network have previously been related to conditioned fear behavior [21], analyses were confined to the entire CS+ (10 s) and furthermore to data segments during which the animals displayed freezing or non-freezing while exposed to the CS+ in R1, R6 and E.

**Figure 4:**
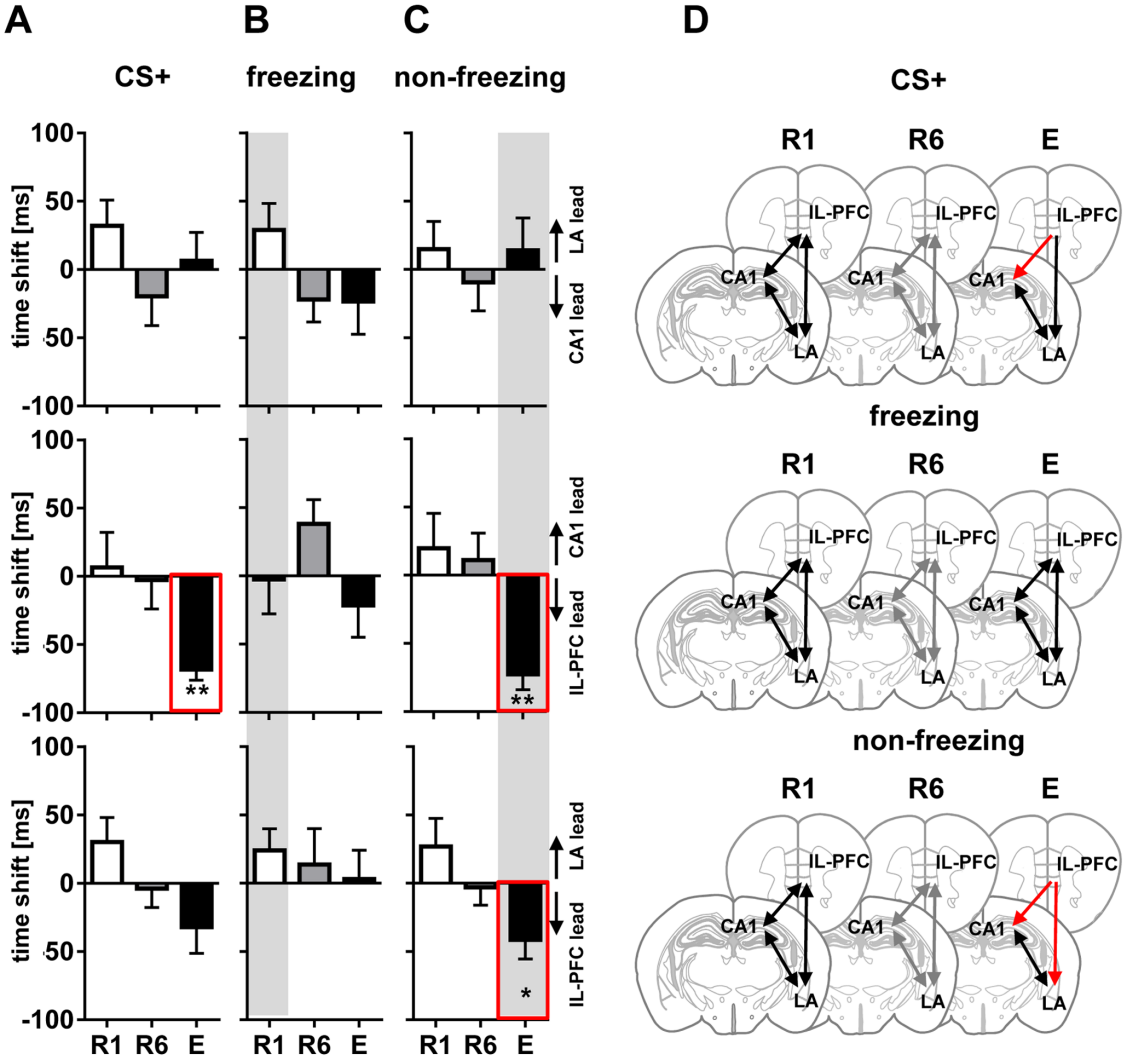
Directional phase locking of LFP theta. Directional phase locking of LFP theta at first CS+ presentation (A), freezing (B) and non-freezing (C) stages during fear memory retrieval (R1; open diagrams), extinction training (R6; grey diagrams), and recall of fear extinction (E; black diagrams). Diagrams indicate time shifts of correlation peaks of LFP theta amplitudes obtained between all possible pairs of recorded regions (CA1/LA, upper row; CA1/IL-PFC, middle; LA/IL-PFC, bottom) as an indicator of the directionality (lagging or leading) between oscillating signals. Positive values indicate that interactions are driven by one region, and negative values indicate that the interaction is driven by the respective other. Note lack of theta directionality during entire CS+ at R1 and R6, and during freezing at R1, R6 and E. Whereas IL-PFC leads to CA1 during CS+ and IL-PFC leads to CA1 and LA during non-freezing at E (highlighted by red lines in A and C). Further single unit phase-locking analyses (see Figure 4 and 5) focused on R1 freezing (B) and non-freezing in E (C) representing the most prominent changes of fear behavior expression as well as LFP based shifts in directionality (grey background). (D) Schematic representation of LFP theta directionality in CA1, LA, and IL-PFC during entire CS+ presentation, freezing and non-freezing at R1, R6 and E. Black and grey lines with two arrowheads indicate high theta synchrony (with no significant time lead or lag), red lines indicate theta directionality. Values are mean±SEM; asterisks indicate the significance level revealed by Wilcoxon Signed Rank test : *,p<0.05; **,p<0.01.

Cross-correlation of instantaneous LFP amplitudes revealed no significant lead or lag in CA1/LA/IL-PFC network during CS+ in R1 and R6, implying that the network is phase synchronized during retrieval of fear (R1) and after extinction training (R6) [Figure 4A; R1: non-significant (ns) lag of CA1 compared to LA (32±18.9 ms), ns lag of IL-PFC compared to CA1 (6.5±25.8 ms), ns lag of IL-PFC compared to LA (30.25±17.9 ms); R6: ns lead of CA1 compared to LA (−19.67±21.4 ms), ns lead of IL-PFC compared to CA1 (−2.6±21.3 ms), ns lead of IL-PFC compared to LA (3.87±13.9 ms); Wilcoxon signed rank test]. During E, no time shift was observed between CA1 and LA and IL-PFC and LA [Figure 4A; E: ns lag of CA1 compared to LA (6.33±20.9 ms), ns lead of IL-PFC compared to LA (−2.6±21.3 ms), ns lead of IL-PFC compared to LA (−32.13±19.2 ms)]. Whereas activity shifted to a theta-driven lead of the IL-PFC compared to CA1 [IL-PFC compared to CA1 (−68.3±7.8 ms), p<0.01].

During freezing, cross-correlation of instantaneous LFP amplitudes revealed no significant lead or lag in CA1/LA/IL-PFC, implying that the network is phase synchronized in the theta frequency range during stages of high fear [Figure 4B; R1: ns lag of CA1 compared to LA (28.9±19.6 ms), ns lead of IL-PFC compared to CA1 (−2.6±25.1 ms), ns lag of IL-PFC compared to LA (24.1±15.7 ms); R6: ns lead of CA1 compared to LA (−22±16.5 ms), ns lag of IL-PFC compared to CA1 (38±17.9 ms), ns lag of IL-PFC compared to LA (3±21.1 ms); E: ns lead of CA1 compared to LA (23.5±23.9 ms), ns lead of IL-PFC compared to CA1 (−21.6±23.3 ms), ns lag of IL-PFC compared to LA (3.0±21.2 ms)].

Next, we analyzed the LFP-based data during stages of low fear expression (non-freezing). Data are illustrated in Figure 4C. Non-freezing stages during R1 and R6 revealed no significant lead or lag of theta between any of the recorded regions in the tripartite circuit [Figure 4C; R1: ns lag of CA1 compared to LA (14.9±20.3 ms), ns lag of IL-PFC compared to CA1 (−9.4±25.6 ms), ns lag of IL-PFC compared to LA (26.9±20.6 ms); R6: ns lead of CA1 compared to LA (−9.4±20.7 ms), ns lag of IL-PFC compared to CA1 (11±19.9 ms), ns lead of IL-PFC compared to LA (−3±13.1 ms)]. During E, activity shifted to a theta-driven lead of the IL-PFC. Theta oscillation of IL-PFC significantly led theta activity of CA1 and LA [IL-PFC compared to CA1 (−72.6±11.4 ms), p<0.01; IL-PFC compared to LA (−41.4±14.0 ms, p<0.05]. No time shift was observed between CA1 and LA theta during non-freezing, implying a phase synchronized state [Figure 4C; ns lag of CA1 compared to LA (14.11±23.7 ms)].

These results indicate that theta oscillations in the tripartite circuit displayed a phase synchronized network upon CS+ presentation during fear memory retrieval and extinction learning, which shift to a significant IL-PFC lead during recall of fear extinction. More specifically, at high fear states (freezing) the CA1/LA/IL-PFC network was highly phase-synchronized throughout the experimental protocol, and it shifted to IL-PFC lead with respect to CA1 and LA during low fear states (non-freezing) upon successful recall of fear extinction during E (Figure 4D).

Next, we analyzed the single cell correlates of this shift in theta synchrony using unit recordings. The focus was on freezing in R1 and non-freezing in E, as significant shifts in LFP-based theta across areas occurred at these behaviors during the respective sessions (Figure 4B and C, marked in grey).

**Figure 5:**
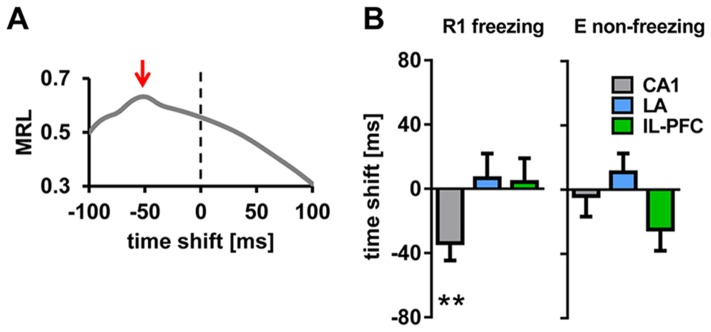
Phase-locking of single unit activity to LFP theta *within* regions. (A) Representative example of directional unit phase-locking analysis of an identified CA1 unit (same unit as in Figure 3B). Mean resultant length vector (MRL), computed by assigning each spike of the unit to the simultaneously recorded LFP theta phase, calculated for 50 different temporal offsets (4 ms bin, from −100 ms to +100 ms), indicate phase lead of the unit by approximately −50 ms (marked by red arrow). (B) Summary of phase-locking analysis of units and LFP theta in CA1, LA and IL-PFC, during freezing in R1 and non-freezing in E. Plotted are mean time shifts between unit activity and the LFP theta phase calculated from MRLs in each area. Note phase locking of units to LFP theta within each region during both freezing and non-freezing, except for phase lead of CA1 units (−33.9±10.7 ms during R1 freezing). Values are mean±SEM; asterisks indicate the significance level revealed by Wilcoxon Signed Rank test : **,p<0.01.

Single unit activity was related to the LFP *within* each recorded region, and *across* regions in two steps of the analysis. *Within* each region, directional unit phase-locking analysis for time shifted local LFPs (representative example in Figure 5A) revealed that neurons of LA and IL-PFC were phase-locked to the local theta rhythm, during both freezing in R1 and non-freezing in E while exposed to the CS+ (Figure 5B). Unit activity in CA1 displayed a phase lead of −33.8±10.7 ms during freezing in R1, while being phase-locked during non-freezing states in E (Figure 5B).

**Figure 6:**
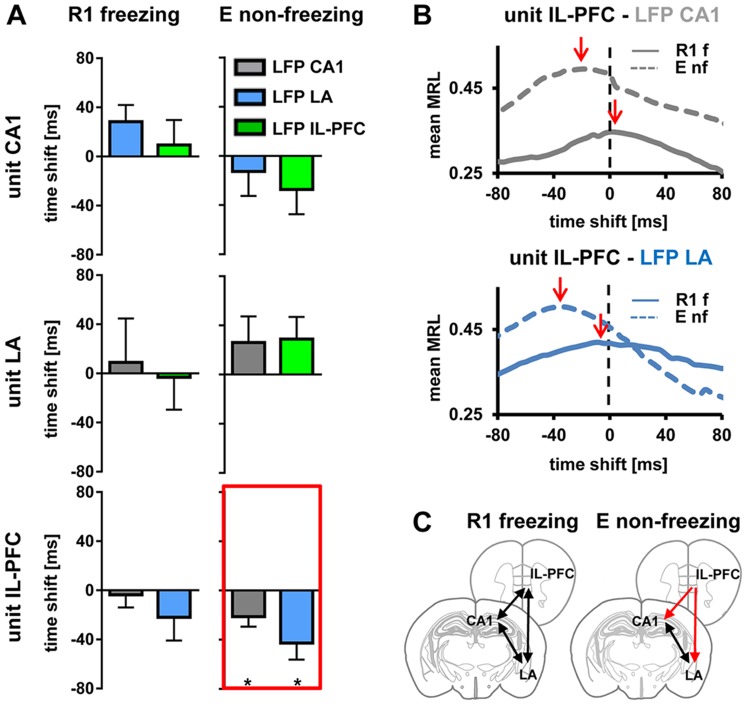
Phase-locking of single unit activity to LFP theta *across* regions. (A) Summary of phase-locking analysis during freezing states in R1 and non-freezing states in E. Each identified unit in CA1 (upper), LA (middle), and IL-PFC (bottom) was assigned a LFP theta phase recorded simultaneously in the other two brain areas (grey, blue, green diagrams indicating LFP in CA1, LA, IL-PFC, respectively). Plotted are mean time shifts between unit activity and the LFP theta phase calculated from MRLs in each area. Note the significant lead of unit activity in IL-PFC related to the LFP theta in CA1 and LA during non-freezing in E (highlighted by red lines). (B) Mean MRL of all identified theta phase-locked IL-PFC units during freezing in R1 and non-freezing in E related to LFP theta oscillations recorded simultaneously in CA1 (upper diagram) and LA (lower diagram). Note the peak MRL (indicated by red arrows) signaling a lead of unit activity in IL-PFC related to LFP theta in CA1 and LA during non-freezing in E. (C) Schematic representation of phase-locking of single unit activity to LFP theta across regions, during freezing at R1 and non-freezing at E. Black lines with two arrowheads indicate high theta synchrony (with no significant time lead or lag), red lines indicate theta directionality. Values are mean±SEM; asterisks indicate the significance level revealed by Wilcoxon Signed Rank test : *,p<0.05. MRL, mean resultant length vector; f, freezing; nf, non-freezing.

*Across* regions, activity of each identified unit was assigned a LFP theta phase recorded simultaneously in the other two brain areas (unit CA1 to LFP LA and IL-PFC, unit LA to LFP CA1 and IL-PFC, unit IL-PFC to LFP CA1 and LA) and directional unit phase-locking analysis was performed on significantly phase-locked units. Results are presented in Figure 6 and Table 1. During freezing stages in R1, no significant lag or lead of the identified units could be observed between any of the recorded regions (Figure 6A and B). Non-freezing stages during E displayed a significant lead of IL-PFC units related to both CA1 and LA LFP theta activity (−21.3±7.7 ms, p<0.05; −42.7±13.5 ms, p<0.05, respectively, Wilcoxon signed rank test; Figure 6A and B). For CA1 and LA unit activity, no significant time shift could be detected during freezing and non-freezing stages in R1 and E (Figure 6A). These changes in theta phase locking obtained from averages of recordings in a total of 13 mice were also apparent from activity patterns in individual animals (Figure S3).

**Table 1:** Summary of directional unit phase-locking analysis.

	R1 freezing			E non-freezing		
Unit CA1	n [unit/animal]	mean ± SEM	p value	n [unit/animal]	mean ± SEM	p value
LFP CA1	13/6	−33.8±10.7	p<0.01	11/8	−4.0±12.7	n.s.
LFP LA	12/5	28.3±13.6	n.s.	6/3	−12.7±19.7	n.s.
LFP IL-PFC	11/6	9.5±20.2	n.s.	5/4	−27.2±20.1	n.s.
**Unit LA**						
LFP LA	6/6	6.7±15.2	n.s.	10/6	10.8±11.8	n.s.
LFP CA1	4/3	9.0±35.7	n.s.	8/5	26.0±21.2	n.s.
LFP IL-PFC	5/3	−3.2±26.3	n.s.	5/4	28.8±18.1	n.s.
**Unit IL-PFC**						
LFP IL-PFC	16/7	4.2±14.7	n.s.	11/8	−24.2±8.1	n.s.
LFP CA1	12/6	−3.7±10.2	n.s.	16/7	−21.3±7.7	p<0.05
LFP LA	6/4	−22.0±18.8	n.s.	6/5	−42.7±13.5	p<0.05

Each identified unit (CA1, LA, IL-PFC) was assigned a theta phase of LFPs recorded in the same and the other two brain areas (unit CA1 - LFP CA1, LA and IL-PFC, unit LA - LFP LA, CA1 and IL-PFC, unit IL-PFC – LFP IL-PFC, CA1 and LA), and lag or lead of the respective unit was determined by the peak MRL value. N [unit/animal] describes the total number of identified phase-locked units and the number of animals in which these units were identified. Results obtained at freezing during retrieval of fear memory (R1), and non-freezing during recall of fear extinction (E). A negative mean [ms] describes a preceding (lead) of the unit, phase-locked to the respective LFP, a positive mean circumscribes the opposite (lag). The p value determines the significance of directionality during R1 and E by use of paired Wilcoxon’s signed rank non-parametric test.

These results indicate that units are phase-locked to LFP theta across regions in CA1/LA/IL-PFC during freezing at fear memory retrieval, whereas theta interactions are directional with IL-PFC activity leading that in CA1 and LA during non-freezing at extinction recall (Figure 6C).

## Discussion

Our findings show regional and directional specificities of theta coherence in circuits involving the infralimbic region of the prefrontal cortex, the hippocampal CA1 and the lateral nucleus of the amygdala, that correlate with individual fear responses during stages of fear memory and extinction. LFP based data revealed that the CA1/LA/IL-PFC network was highly phase-synchronized at high fear states (freezing), and it shifted to IL-PFC lead with respect to CA1 and LA during low fear states (non-freezing) upon successful recall of fear extinction during E. Single cell correlates closely resembled LFP based dynamics. *Within each region*, the firing of a significant fraction of neurons was phase-locked to the local theta rhythm upon CS+ presentation, with unit activity in CA1 displaying a phase lead of 30 ms during high fear states. *Across regions,* theta coherence and directionality correlated with individual behavioral responses to the fear-conditioned stimulus. Upon CS+ evoked freezing, units were phase-locked to synchronized theta oscillations in CA1/LA/IL-PFC pathways, characterizing states of fear memory retrieval. When CS+ presentations evoked no fear during extinction recall, theta interactions were directional with prefrontal cortical activity leading hippocampal and amygdalar activity, and CA1 and LA theta oscillations were phase-locked to IL-PFC units at a delay of approximately 20–40 ms.

It previously has been shown that the amygdalo-hippocampal-prefrontal network exhibits an increase in theta synchrony during CS+ presentations after fear conditioning, an overall decline in theta synchrony during extinction learning, and a theta rebound particularly involving the IL-PFC during extinction recall [5], [21], [25]–[27], [33]. However, because conclusions on changes in theta activity were derived from LFP recordings, the contribution of subpopulations of neurons and their regional or directional specificity could not be disentangled. Furthermore, the correlation with behavioral states of high fear and extinguished fear remained elusive. A subsequent study reported that changes in theta coherence in these circuits during REM sleep are correlated with inter-individual variations in fear memory consolidation [8]. The percentage of neurons that were significantly phase-locked to the local REM theta rhythm was at around 10–20% in BLA and mPFC [8] thereby matching the fraction of theta phase-locked units observed during CS+ presentation in the present study. Remarkably, REM theta coherence that correlated with the success of fear memory consolidation displayed directionality in hippocampus-to-BLA and BLA-to-mPFC pathways [8], an effect largely mirroring the directional theta coherence in IL-PFC-to-hippocampus and IL-PFC-to-LA circuits that correlated with the lack of fear responses upon extinction recall (present study). An anterograde tracing study in a transgenic rat model, in which neurons express a dendritically targeted PSD-95:Venus fusion protein under the control of a *c-fos* promoter, has revealed that the dominant input to active neurons in the LA during retrieval of fear extinction originates from the infralimbic region of the mPFC, whereas the retrieval of fear memory is associated with greater hippocampal and prelimbic mPFC inputs [34]. In view of the role of the IL-PFC for consolidation and recall of fear extinction [14], [35], [36], these results let us suggest that directional theta coherence is a crucial mechanism in entraining amygdalar and hippocampal neuronal discharges to infralimbic prefrontal cortical activity during states of successfully extinguished fear. The following line of findings is supportive of this conclusion.

An increase in theta coherence has previously been proposed to coordinate dorsal hippocampal-prefrontal cortical interactions in a spatial memory task [37]. Phase locking in these interactions occurred at 50 ms [32], at a range resembling that in CA1/LA/IL-PFC pathways during extinction recall. Although prefrontal cortical units were delayed and suggested to be recruited by hippocampal theta oscillations in that previous study [32], as opposed to a leading role of the IL-PFC in extinction-related theta, these findings suggest a convergent set of mechanisms guiding interactions in these pathways. Directionality of IL-PFC to LA interactions has also been inferred from field potential recordings in 5-HT transporter-deficient mice, with a shift from IL-PFC to LA lead relating to a delay in fear extinction upon experience of social defeat [27]. In any case, the evidence for prefrontal cortical involvement in amygdalar and hippocampal phase-locking is only correlative, and identification of the underlying circuit mechanisms is an important open issue.

Recent findings showed that IL-PFC neurons form monosynaptic connections to intercalated GABAergic cells in the amygdala, which mediate feed-forward inhibition to principal neurons in LA and central amygdala and this pathway is crucial for fear extinction [38]–[43]. This fact suggests the intriguing possibility that theta phase locking in the amygdala during extinction recall might arise via the GABAergic inhibitory network. While recruitment of intercalated GABAergic cells to theta oscillations has not been reported yet, the firing of calbindin-positive GABAergic interneurons in BLA is precisely modulated by the hippocampal theta rhythm [44], and parvalbumin-positive GABAergic interneurons in this area have a preponderance to discharge at theta frequencies and to theta-lock synaptically connected principal neurons [45], [46]. Available data thus indicate that GABAergic synaptic influences play an important role in theta synchrony in the basolateral amygdala and connected pathways. Furthermore, distinct populations of neurons have been identified for signaling of fear memory and extinction in the basal and lateral nuclei of the amygdala [47], [48]. It is intriguing to suggest that theta synchrony is an important mechanism for functional organization of the respective neuronal ensembles, with distinct subpopulations of GABAergic neurons mediating the distinct patterns of theta synchrony in connected hippocampal and prefrontal cortical pathways signaling recall of fear memory and extinction.

Changes in theta coherence in amygdalo-hippocampal-prefrontal cortical pathways correlating with fear memory and extinction involved the dorsal hippocampus [8], [21], [25]–[27], [49], matching observations in other memory tasks that neural activity in the IL-PFC synchronizes with theta frequency oscillations in the dorsal hippocampus [32], [37], [50]. Other evidence indicated the existence of theta coherence across dorsal and ventral hippocampal areas, with activity in the ventral but not dorsal hippocampus highly correlating with that in the IL-PFC in anxiogenic environments [9], [10]. Hippocampal pyramidal cells indeed fire in a stable theta phase relationship in both the dorsal and the ventral hippocampus [51], and the hippocampus as a whole exhibits a continuous theta phase progression along its septotemporal axis [52]. In keeping with this, unit activity in the BLA phase-locks to both ventral and dorsal CA1 theta oscillations [44], and the dorsal CA1 represents a more regular and reproducible theta rhythm [44], [53]. A ventral hippocampal circuit gating amygdala-based fear has recently been identified to involve the prelimbic area of the mPFC [36], [54]. Inactivation of the ventral hippocampus increased fear and activity in principal neuron in the prelimbic mPFC in fear-extinguished, but not in fear-conditioned rats, consistent with hippocampal gating of states of low fear after extinction. While the impact of theta oscillations in these pathways remain to be investigated, it is interesting to note that directionally correlated activity between the amygdala and the dorsal anterior cingulate cortex, an area in the primate brain thought to regulate fear expression much resembling the prelimbic mPFC in the rodent brain [55], has been proposed to be a predictive mechanism for the resistance of aversive memories to extinction [22]. Directionality and coherence of neuronal activity may thus represent a principle mechanism to recruit neuronal subpopulations across brain areas into the various stages of a fear memory trace and to gate the respective states of fear and extinction.

## Conclusion

Our data support the view that fear memory and extinction depend on distributed representations in amygdala-hippocampal-prefrontal cortical circuits [4], [20], [34], [54], [56]. More specifically our results indicate that the directional dynamics of theta-entrained activity across these areas guide changes in appraisal of threatful stimuli during fear memory and extinction retrieval: phase-locked theta synchrony signals fear during recall of conditioned fear, while a prefrontal cortical lead in these theta interactions contributes to signaling no fear after extinction. Given that exposure therapy involves procedures and pathways similar to those during extinction of conditioned fear responses in the laboratory [17], one therapeutical extension might be useful that imposes artificial theta activity to prefrontal cortical-amygdalo-hippocampal pathways mimicking the directional lead of the IL-PFC observed during successful extinction recall. In fact, frontal theta oscillations have recently been shown to regulate negative emotions by cognitive reappraisal in humans [57].

## Supporting Information

Figure S1 **Fear conditioning paradigm.** (Day1) During adaptation animals were exposed to six CS− only and the entire session was repeated six hours later. (Day2) Conditioning took place on the following day: The CS+ (marked in red) was presented three times, every time co-terminating with an electric footshock. This conditioning session was repeated once 6 h later. Memory was tested on the next 2 days. (Day 3) Six consecutive retrieval sessions were carried out (R1 through R6), with 30 minutes between sessions. (Day 4) Extinction memory was recalled the next day in session E. All retrieval and extinction sessions were identical (see inset), and contained four CS− and four CS+ presentations. Based on recently published data [21] we analysed only the first presented conditioned stimulus (1CS+) of R1, R6 and E (marked by rectangles). These time points displayed the highest (R1 and E) and lowest (R6) degree of theta correlation in a regionally specific manner in the CA1-LA-IL-PFC network [21].
(TIF)

Figure S2 **Theta coherence during fear memory retrieval (R1), after extinction training (R6) and recall of fear extinction** **(E).** Theta coherence during fear memory retrieval (R1), after extinction training (R6), and recall of fear extinction (E) was calculated from pairs of recordings in CA1/LA, CA1/IL-PFC, and IL-PFC/LA in response to presentation of the 1CS+. Note the robust coherence in all recording pairs in the theta frequency range. Coherence was computed as follows: After applying Hanning window and Fourier transformation of LFP signals coherence was performed in a frequency range between 1 and 1000 Hz with a 50% overlap window (Neuroexplorer, Nex Technologies).
(TIF)

Figure S3 **Individual (one animal) example of IL-PFC unit activities phase-locked to LFP theta across and within regions during freezing states in R1 and non-freezing states in E.** Each identified IL-PFC unit was assigned a LFP theta phase recorded simultaneously in IL-PFC (green diagrams) and the other two brain areas (grey and blue diagrams indicating LFP in CA1, and LA, respectively). Data are indicative of a shift in theta directionality towards PFC lead during E non-freezing.
(TIF)
