# Supplementary figures and images for: Directional Theta Coherence in Prefrontal Cortical to Amygdalo-Hippocampal Pathways Signals Fear Extinction
Source: PLoS One. 2013 Oct 24;8(10):e77707. doi: 10.1371/journal.pone.0077707 (PMC3812006; doi:10.1371/journal.pone.0077707)

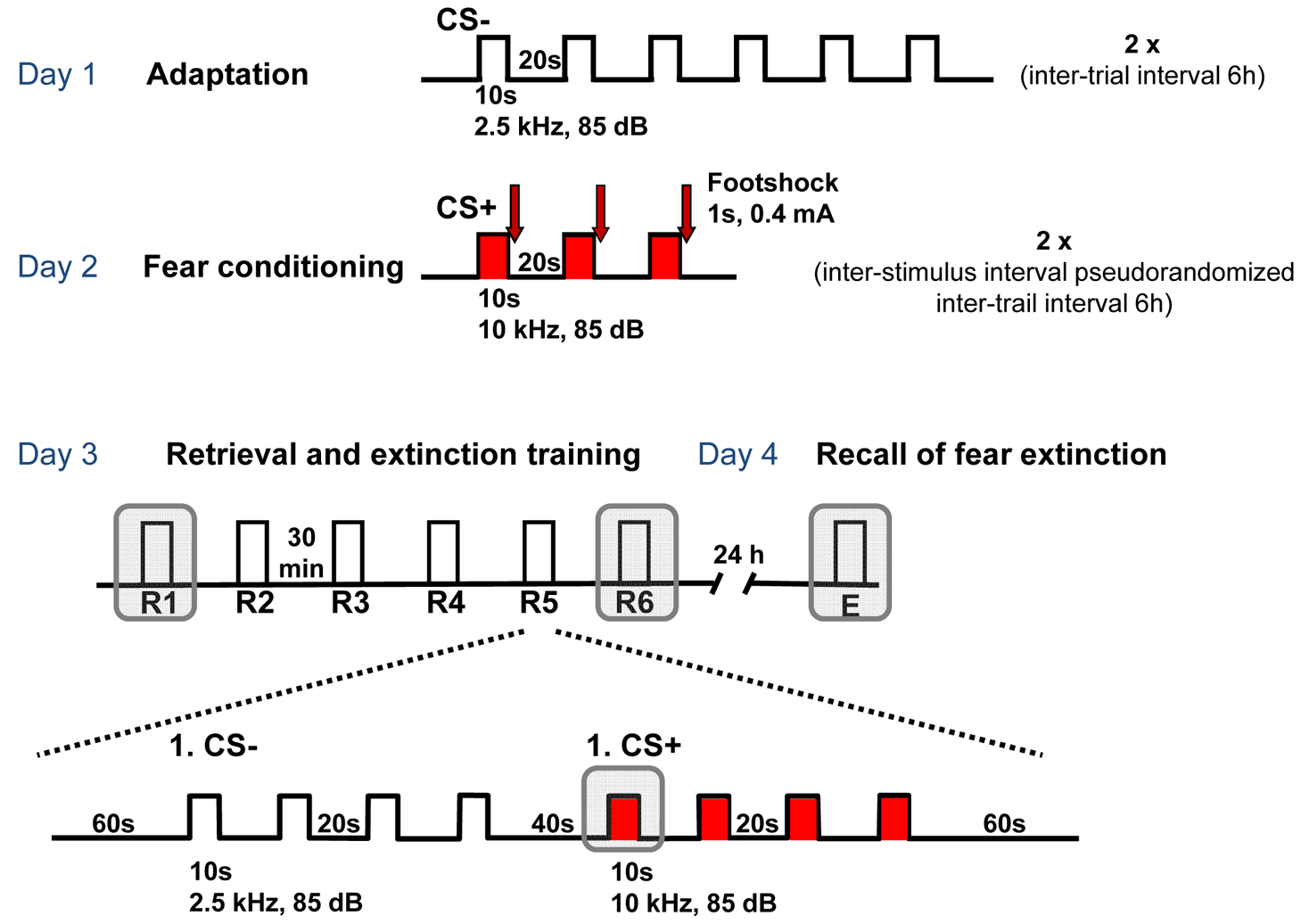

Supplement: Figure S1 — Fear conditioning paradigm. (Day1) During adaptation animals were exposed to six CS− only and the entire session was repeated six hours later. (Day2) Conditioning took place on the following day: The CS+ (marked in red) was presented three times, every time co-terminating with an electric footshock. This conditioning session was repeated once 6 h later. Memory was tested on the next 2 days. (Day 3) Six consecutive retrieval sessions were carried out (R1 through R6), with 30 minutes between sessions. (Day 4) Extinction memory was recalled the next day in session E. All retrieval and extinction sessions were identical (see inset), and contained four CS− and four CS+ presentations. Based on recently published data [21] we analysed only the first presented conditioned stimulus (1CS+) of R1, R6 and E (marked by rectangles). These time points displayed the highest (R1 and E) and lowest (R6) degree of theta correlation in a regionally specific manner in the CA1-LA-IL-PFC network [21]. (TIF) [file pone.0077707.s001.tif]

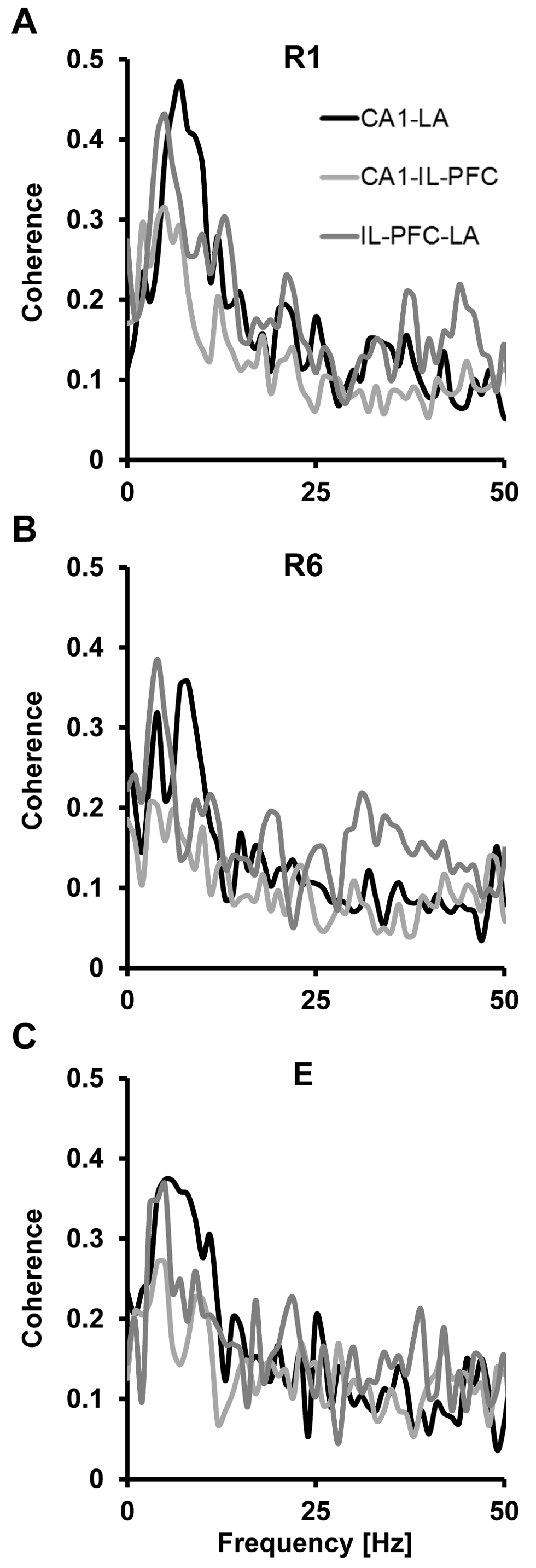

Supplement: Figure S2 — Theta coherence during fear memory retrieval (R1), after extinction training (R6) and recall of fear extinction (E). Theta coherence during fear memory retrieval (R1), after extinction training (R6), and recall of fear extinction (E) was calculated from pairs of recordings in CA1/LA, CA1/IL-PFC, and IL-PFC/LA in response to presentation of the 1CS+. Note the robust coherence in all recording pairs in the theta frequency range. Coherence was computed as follows: After applying Hanning window and Fourier transformation of LFP signals coherence was performed in a frequency range between 1 and 1000 Hz with a 50% overlap window (Neuroexplorer, Nex Technologies). (TIF) [file pone.0077707.s002.tif]

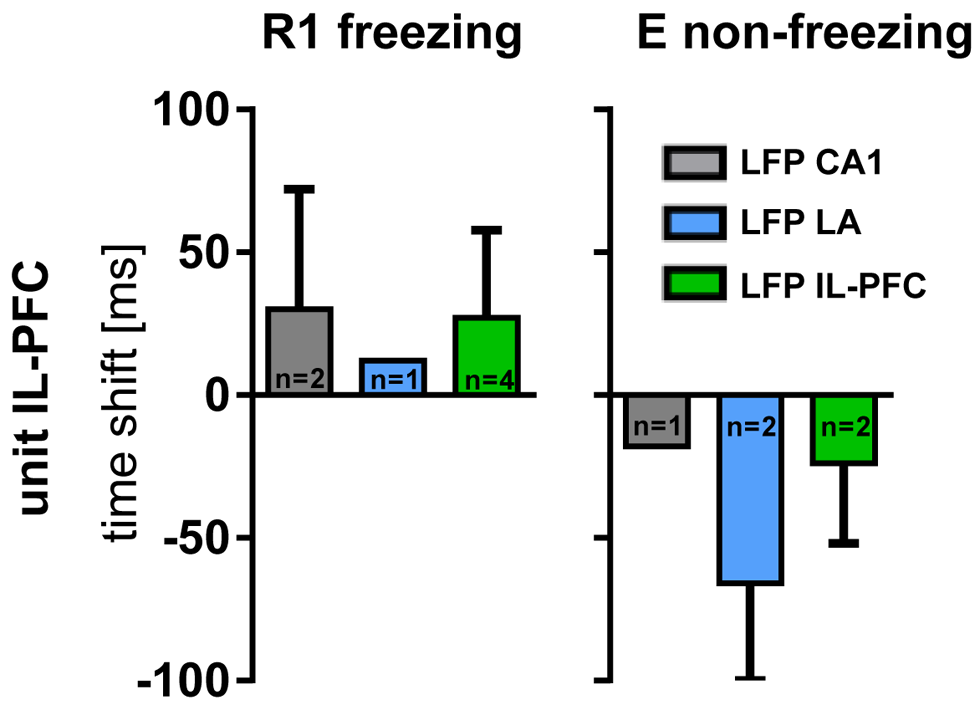

Supplement: Figure S3 — Individual (one animal) example of IL-PFC unit activities phase-locked to LFP theta across and within regions during freezing states in R1 and non-freezing states in E. Each identified IL-PFC unit was assigned a LFP theta phase recorded simultaneously in IL-PFC (green diagrams) and the other two brain areas (grey and blue diagrams indicating LFP in CA1, and LA, respectively). Data are indicative of a shift in theta directionality towards PFC lead during E non-freezing. (TIF) [file pone.0077707.s003.tif]
